# Environmental exposure to organophosphate esters and suspected non-alcoholic fatty liver disease among US adults: A mixture analysis

**DOI:** 10.3389/fpubh.2022.995649

**Published:** 2022-10-20

**Authors:** Haisheng Chai, Weiye Hu, Yaoyao Dai, Xiaohan Zhu, Ping'an Qian, Junfeng Zhu

**Affiliations:** ^1^Department of Hepatology, Shanghai Municipal Hospital of Traditional Chinese Medicine, Shanghai University of Traditional Chinese Medicine, Shanghai, China; ^2^Department of Hepatology, Yueyang Integrated Chinese and Western Medicine Hospital, Shanghai University of Traditional Chinese Medicine, Shanghai, China

**Keywords:** organophosphate esters, non-alcoholic fatty liver disease, adults, weighted quantile sum regression, environmental epidemiology

## Abstract

**Objectives:**

Non-alcoholic fatty liver disease (NAFLD) is the most common chronic liver disease worldwide. We evaluated NAFLD using the US FLI to determine whether there is an association between urinary organophosphorus (OPE) levels and the “prevalence” of NAFLD in US individuals.

**Methods:**

The current study included 1,102 people aged 20 years and older with information from the 2011–2014 U.S. National Health and Nutrition Examination Survey. NAFLD was assessed using the U.S. FLI. Individual OPE metabolites and OPE combinations were linked to NAFLD using logistic regression and weighted quantile sum (WQS) regression. All analyzes were carried out separately on males and females. The possible impacts of age, serum total testosterone (TT), and menopausal state, as well as the importance of the interaction term with exposure, were investigated using stratified analysis.

**Results:**

Bis (2-chloroethyl) phosphate and bis (1,3-dichloro-2-propyl) phosphate were associated with NAFLD in all males after adjusting for covariates (*P* < 0.05). A combination of OPEs (OPE index) was positively linked with NAFLD in the WQS analysis of all males (odds ratio for OPE index: 1.52; 95% CI: 1.06, 2.19). Stratified analyzes for males revealed that considerable connections were largely confined to individuals over 60 years old or with low total testosterone. In women, the connection was limited and inconsistent, except for the OPE index, which was positively linked with NAFLD in post-menopausal women.

**Conclusions:**

In this study, environmental exposure to OPE was linked to an elevated risk of NAFLD in males, particularly those over 60 years old or with low TT levels. Aside from the continuous positive connection of a combination of OPEs with NAFLD risk in post-menopausal women, these correlations were weaker in women. However, these findings should be taken with caution and verified in future investigations by collecting numerous urine samples in advance to strengthen OPE exposure estimates.

## Introduction

The prevalence of non-alcoholic fatty liver disease (NAFLD) has risen with the rising prevalence of obesity in recent years (1), and it is now considered the most prevalent chronic liver disease worldwide (2). NAFLD is a developing issue globally and affects 25% of adults worldwide. It is a group of liver metabolic diseases characterized by lipid buildup in hepatocytes. NAFLD is a complex illness with a wide range of pathology that can lead to more serious disorders. Patients with NAFLD develop non-alcoholic steatohepatitis, which is characterized by cell death, inflammation, and fibrogenesis and eventually leads to cirrhosis and liver cancer (3, 4). Simultaneously, NAFLD has been demonstrated to be a separate risk factor for cardiovascular disease, diabetes, and overall mortality (5). Unless particular diagnostics for NAFLD are available, this disorder is frequently undetected until it progresses and develops into permanent liver damage. As a result, the majority of patients with NAFLD are unaware that they have this dangerous illness. Moreover, current therapy options are restricted, including dietary restrictions and lifestyle adjustments (6), because no FDA-approved medications are available (2). Investigating the risk factors of NAFLD and preventing the illness from occurring are crucial because of the gradual onset of NAFLD and the lack of effective treatment options.

With the elimination of polybrominated diphenyl ethers, organophosphate esters (OPEs) are now widely employed as flame retardants and plasticizers globally. OPE production has grown continuously over the past 15 years (7). OPEs can be found in a variety of products, including electronics, building materials, automobiles, furniture, vehicle seats, plastics, and textiles. As a result, OPEs and their metabolites are now commonly discovered in human urine, blood, placental tissue, and breast milk worldwide (8). Concerns about OPEs have grown in recent years, and previous research revealed that OPEs can have a variety of negative impacts on creatures and people, including poor reproductive health and birth outcomes, obesity, neurodevelopment, hypertension, and sex steroid hormones (9–12). Organophosphate esters (OPEs) have the potential to disrupt endocrine (1). Exposure to OPEs can affect homeostasis, and disturbance of homeostasis may lead to adverse health outcomes (2, 3). In addition, a diverse variety of novel alternative flame retardants (nFRs) were continuously discovered at increasing concentrations in environmental and human matrices. Evidence showed that nFR exposure is linked to endocrine system disruptions, which are related to the pathophysiology of several metabolic diseases, including NAFLD. NAFLD is a multifactorial illness defined by an unregulated buildup of fats (lipids) in liver cells and is implicated in various etiologies, including occupational and environmental chemical exposure (13). A number of risk factors, including exposure to environmental toxicants, play a role in the development and progression of NAFLD. Environmental factors may contribute to the development and progression of NAFLD through a variety of biological alterations, including mitochondrial dysfunction, reactive oxygen species production, nuclear receptor dysregulation, and disruption of inflammatory and immune-mediated signaling pathways (4–6). Furthermore, environmental contaminants might alter immune system components and hence influence immunological responses and disease susceptibility (4).

Although various *in vitro* and *in vivo* studies have investigated the negative health effects of OPE exposure (7–9), human data results on adult OPEs and NAFLD are still relatively scarce. Given the prevalence of OPEs in the environment and their potential deleterious consequences, investigating their impact on the incidence and progression of NAFLD is vital. The goal of this study was to look at the link between five urine OPE metabolites and NAFLD in adult participants of U.S. National Health and Nutrition Examination Survey (NHANES) 2011–2014 and to look into any potential interactions.

## Methods

### Study participants

The National Health and Nutrition Examination Survey (NHANES), a unique national data source on the health and nutritional status of the U.S. population, is conducted by the National Center for Health Statistics (NCHS; Hyattsville, MD, USA) and relies on a stratified multistage probability sample based on selection counties, neighborhoods, households, and household insiders to represent the civilian, non-institutionalized U.S. population. Data were gathered by interviews, standardized exams, and the collection of biospecimens (14). Survey participants were asked to complete a series of questions in a detailed home interview before going to a Mobile Examination Center (MEC) as planned. At the MEC, they undergo physical examinations to collect anthropometric and health data and biological specimens for laboratory testing (e.g., nutritional status, health, and environmental exposures) (15). The study sample included 11,326 people over the age of 20 from two consecutive NHANES cycles (2011–2012, 2013–2014). Individuals without baseline data on DPHP, BDCPP, BCPP, BCEP, and DBUP concentrations, as well as age, gender, ethnicity, and BMI, were eliminated. The analysis eventually comprised 1,102 individuals with full outcomes and related collaboration factors.

### Organophosphate ester metabolites

In 0.4 ml of spot urine from environmental subsample C (a random 1/3 sample of people aged 6 years and older), nine urinary OPE metabolites were detected. After combining the data from both cycles, five OPE metabolites with final detection frequencies more than 50% were included in the analysis: DPHP, BDCPP, BCPP, BCEP, and DBUP. The lower limit of detection (LLOD) of the five OPEs metabolites in the 2011–2012 cycle was all 0.10 g/L. LLOD for BCEP was 0.08 g/L, 0.16 g/L for DPHP, 0.10 g/L for BCPP, 0.11 g/L for BDCPP, and 0.05 g/L for DBUP during the 2013–2014 cycle. According to the NHANES Analysis Guidelines (cdc.gov/nchs/nhanes/2013–2014/SSFLRT_H.htm), replace the values below LLOD with LLOD/root 2.

### NAFLD assessment

Although liver biopsy is widely accepted as the gold standard method for detecting NAFLD, its invasiveness makes it unsuitable for community research. In this investigation, we employed another indicator developed by Ruhl and Everhart for determining the presence of NAFLD, the US FLI, which has been shown to be more accurate than the Fatty Liver Index in the US population. The relevant literature states that when FLI < 30 excludes fatty liver, the specificity is 52% (sensitivity 83%). FLI ≥60 determines fatty liver with a sensitivity of 69%, (specificity 77%) (16). Race/ethnicity, age, waist circumference, GGT, rapid insulin, and glucose are all used to generate the US FLI:

### Covariates

The multivariate model included latent variables confounding OPEs and NAFLD (Supplementary Figure S1), such as gender (male/female), age (continuous variable), education level, race/ethnicity, marital status, family poverty income ratio (1, 1–3, >3), BMI (Underweight/Normal weight, overweight, and obese), total cholesterol (continues), and creatinine (continues). Smoking status was divided into Never, ever (quit smoking cigarettes at least 1 day), and current (now smoke and at least 100 cigarettes in life). Hypertension was described as having a systolic blood pressure more than 140 mmHg or a diastolic blood pressure greater than 90 mmHg. diabetes (yes/no), alcohol intake (yes/no), physical activity (yes/no). Also, Supplementary Table S9 showed the Pearson correlation matrix (coefficients) between the selected covariates and NAFLD.

### Statistical analysis

Male and female demographic data were expressed as mean (standard deviation, SD) or median (interquantile range, IQR) of continuous variables and proportions of categorical components based on NAFLD status. Natural log transformation (ln transformation) was used for urine creatinine and OPE metabolites because they are usually right skewed.

Regression models were separately constructed for males and females because of the well-documented gender inequalities in metabolic disorders. We initially utilized logistic regression to look at the relationship between certain OPE metabolites and NAFLD. OPE metabolites were modeled as continuous (ln-transformed) and categorical variables in regression analysis. Metabolites detected in 80% of individuals were classified into quartiles, with the first quartile serving as the reference, whereas tertiles were utilized for metabolites detected in 80% of participants. The categorical OPE metabolites were modeled as ordinal variables for linear trend testing. *P* values were adjusted using the Benjamini-Hochberg false discovery rate (FDR) correction to adjust for multiple testing.

We also utilized weighted quantile sum (WQS) regression to assess the combined effect of various OPE metabolites. WQS is a multi-pollutant model that has been widely used to investigate the combined effects of a variety of environmental pollutants (17). WQS allows the examination of the overall impact of chemical mixtures while simultaneously allowing for collinearity and identifying the major chemical species responsible for the observed connections. Simulations suggest that WQS outperforms approaches that focus on forecasting performance in association analysis. In this work, we created a WQS index of OPE based on metabolite quartiles (OPE index) and ran a WQS regression analysis by gender with 500 bootstrap samples in each dataset. We performed two sets of WQS regression analyzes because we did not know which OPE metabolite was linked to NAFLD. One set assumed that all components of the WQS index are positively correlated with NAFLD, whereas the other assumed that all components of the index are negatively correlated with NAFLD. The computed WQS index regression coefficients were interpreted as the mean change in log probabilities of NAFLD for each increasing index quartile.

Furthermore, we explored the relevance of the interaction term in multivariate models and stratified the results for age, serum testosterone levels, and menopausal status (women) (exposure variable effect-adjusted variable). NAFLD was related to age, and OPE exposure changed with age; therefore, analyzes were stratified further by age (< 60 and ≥60 years). Although epidemiological evidence is currently limited, laboratory investigations have shown that OPE has an antiandrogenic impact. Additionally, testosterone with various mechanisms of action may be linked to NAFLD in men and women. We expected that testosterone would change the effects of OPE. Serum total testosterone (TT) values were employed as they are the only androgens assessed in NHANES 2011–2014. TT was categorized as “low” or “high” based on their respective medians (426 ng/dl for males and 19.26 ng/dl for women).

A stratified analysis of menopausal status was also performed in women because of the unique risk profiles for metabolic dysfunction before and after menopause. We also ran several sensitivity studies to examine the robustness of our primary findings. First, the Bayesian kernel machine regression (BKMR) method was used to further validate the association of mixed exposure to OPE with NAFLD. Kernel machine regression is a common tool in machine learning that can flexibly model the relationship between a large number of variables by mapping or projecting one data series to another in a one-to-one manner to produce specific results (18). In contrast, Bayesian kernel machine regression (BKMR) is a new method with non-parametric Bayesian and statistical learning capabilities that allows flexible study of the combined effects of environmental mixtures on human health (19, 20). It can be used in that study to test (1) the relationship between different OPEs and the risk of NAFLD; and (2) the overall impact of OPEs. Second, the data were reanalyzed using the sampling weight to evaluate the consistency of the primary results. These studies were confined to classic binary logistic regression because the WQS approach does not enable survey designs.

The results of the regression analysis are presented as odds ratios (ORs) and 95% confidence intervals (CIs). All analyzes were performed in R version 3.4 (Foundation, Vienna, Austria).

## Results

### Baseline characteristics

This study included 1,102 individuals, including 355 patients with NAFLD. Table 1 summarizes the demographic characteristics and biochemical measurements based on gender and NAFLD status. Notably, those with NAFLD were more likely to be older, male, obese, diabetic, and hypertensive than those without NAFLD. The patients with NAFLD had increased levels of triglycerides, fasting insulin, alanine transaminase, aspartate transaminase, gamma glutamyl transferase, and Homeostatic Model Assessment for Insulin Resistance score (*P* < 0.05). Furthermore, remarkable baseline variations in race/ethnicity, education, marital status, poverty–income ratio (PIR), smoking, physical activity, waist circumference, and high-density lipoprotein–cholesterol levels.

**Table 1:** Baseline characteristics of the 1,102 participants.

	**Male (*****N*** = **541)**		**Female (*****N*** = **561)**		**Total population (*****N*** = **1,102)**		
	**No NAFLD (*N* = 342)**	**NAFLD (*N* = 199)**	**No NAFLD (*N* = 405)**	**NAFLD (*N* = 355)**	**No NAFLD (*N* = 747)**	**NAFLD (*N* = 355)**	**Total**
Age [median (IQR)]	45.0 (31.0, 58.0)	54.0 (40.0, 66.0)	47.0 (33.0, 63.0)	56.0 (42.0, 65.0)	46.0 (32.0, 61.0)	54.0 (41.0, 66.0)	49.0 (34.0, 63.0)
**BMI Category [*****n*** **(%)]**							
Underweight/normal weight	145 (42.4)	4 (2.0)	160 (39.5)	0 (0.0)	305 (40.8)	4 (1.1)	309 (28.0)
Overweight	155 (45.3)	59 (29.6)	141 (34.8)	37 (23.7)	296 (39.6)	96 (27.0)	392 (35.6)
Obese	42 (12.3)	136 (68.3)	104 (25.7)	119 (76.3)	146 (19.6)	255 (71.9)	401 (36.4)
**Poverty income ratio [*****n*** **(%)]**							
PIR ≤ 1	70 (20.5)	43 (21.6)	80 (19.8)	38 (24.4)	150 (20.1)	81 (22.8)	231 (21.0)
1 < PIR ≤ 3	125 (36.5)	88 (44.2)	161 (39.8)	73 (46.8)	286 (38.3)	161 (45.4)	447 (40.6)
3 < PIR	147 (43.0)	68 (34.2)	164 (40.5)	45 (28.8)	311 (41.6)	113 (31.8)	424 (38.4)
**Marital status [*****n*** **(%)]**							
Married/cohabiting	202 (59.1)	135 (67.8)	233 (55.1)	87 (55.8)	425 (56.9)	222 (62.5)	647 (58.7)
Widowed/divorced/separated	53 (15.5)	43 (21.6)	97 (24.0)	47 (30.1)	150 (20.1)	90 (25.4)	240 (21.8)
Never married	87 (25.4)	21 (10.6)	85 (21.0)	22 (14.1)	172 (23.0)	43 (12.1)	215 (19.5)
**Education [*****n*** **(%)]**							
Under high school	76 (22.2)	42 (21.1)	65 (16.0)	44 (28.2)	141 (18.9)	86 (24.2)	227 (20.6)
High school or equivalent	84 (24.6)	48 (24.1)	67 (16.5)	33 (21.2)	151 (20.2)	81 (22.8)	232 (21.1)
Above high school	182 (53.2)	109 (54.8)	273 (67.4)	79 (50.6)	455 (60.9)	188 (53.0)	643 (58.3)
**Race/ethnicity [*****n*** **(%)]**							
Mexican American	31 (9.1)	37 (18.6)	37 (9.1)	32 (20.5)	68 (9.1)	69 (19.4)	137 (12.4)
Other hispanic	29 (8.5)	22 (11.1)	36 (8.9)	15 (9.6)	65 (8.7)	37 (10.4)	102 (9.3)
Non-hispanic white	161 (47.1)	102 (51.3)	180 (44.4)	74 (47.4)	341 (45.7)	176 (49.6)	517 (46.9)
Non-hispanic black	66 (19.3)	17 (8.5)	91 (22.5)	20 (12.8)	157 (21.0)	37 (10.4)	194 (17.6)
Other/multi-racial	55 (16.1)	21 (10.6)	61 (15.1)	15 (9.6)	116 (15.5)	36 (10.2)	152 (13.8)
**Smoking status [*****n*** **(%)]**							
Never	154 (45.0)	85 (42.7)	269 (66.4)	95 (60.9)	423 (56.6)	180 (50.7)	603 (54.7)
Ever	99 (28.9)	74 (37.2)	64 (15.8)	40 (25.6)	163 (21.8)	114 (32.1)	277 (25.1)
Current	89 (26.0)	40 (20.1)	72 (17.8)	21 (13.5)	161 (21.6)	61 (17.9)	222 (20.2)
**Alcohol [*****n*** **(%)]**							
No	52 (15.2)	25 (12.6)	134 (33.1)	61 (39.1)	186 (24.9)	86 (24.2)	272 (24.7)
Yes	290 (84.8)	174 (87.4)	271 (66.9)	95 (60.9)	561 (75.1)	269 (75.8)	830 (75.3)
**Physical activity [*****n*** **(%)]**							
No	133 (38.9)	124 (62.3)	192 (47.4)	92 (59.0)	325 (43.5)	216 (60.9)	541 (49.1)
Yes	209 (61.1)	75 (37.7)	213 (52.6)	64 (41.0)	422 (56.5)	139 (39.1)	561 (50.9)
**Hypertension [*****n*** **(%)]**							
No	245 (71.6)	100 (50.3)	286 (70.6)	59 (37.8)	531 (71.1)	159 (44.8)	690 (62.6)
Yes	97 (28.4)	99 (49.7)	119 (29.4)	97 (62..2)	216 (28.9)	196 (55.2)	412 (37.4)
**Diabetes [*****n*** **(%)]**							
No	325 (95.0)	152 (76.4)	379 (93.6)	111 (71.2)	704 (94.2)	263 (74.1)	967 (87.8)
Yes	17 (5.0)	47 (23.6)	26 (6.4)	45 (28.8)	43 (5.8)	92 (25.9)	135 (12.2)
**Urinary creatinine [median (IQR)]**	117.0 (74.0, 174.0)	129.0 (96.0, 184.0)	79.0 (47.0, 127.0)	108.0 (62.0, 162.0)	95.0 (56.0, 154.0)	126.0 (81.0, 175.0)	105.0 (62.0, 161.0)
**Waist circumference (cm) [median (IQR)]**	93.3 (85.2, 100.0)	112.0 (104.0, 122.0)	90.8 (81.8, 98.3)	114.0 (103.0, 123.0)	92.1 (84.1, 99.8)	112.0 (103.0, 122.0)	97.3 (87.3, 108.0)
**Total cholesterol (mg/dl) [median (IQR)]**	183.0 (161.0, 208.0)	182.0 (154.0, 210.0)	191.0 (165.0, 219.0)	193.0 (164.0, 223.0)	187.0 (163.0, 213.0)	187.0 (158.0, 217.0)	187.0 (162.0, 214.0)
**Triglycerides (mg/dl) [median (IQR)]**	89.0 (66.0, 122.0)	146.0 (96.5, 205.0)	81.0 (59.0, 117.0)	134.0 (102.0, 181.0)	85.0 (62.0, 118.0)	141.0 (99.5, 194.0)	100.0 (69.0, 147.0)
**HDL-cholesterol (mg/dl) [median (IQR)]**	50.0 (43.0, 59.0)	40.0 (35.0, 47.0)	60.0 (51.0, 71.0)	50.0 (41.0, 57.0)	55.0 (46.5, 65.0)	44.0 (37.0, 52.0)	51.0 (42.0, 62.0)
**Fasting insulin (pmol/L) [median (IQR)]**	39.7 (27.1, 56.9)	107 (80.6, 162)	44.1 (31.0, 63.3)	121 (95.1, 170)	41.9 (29.0, 60.1)	115 (86.8, 167)	56.9 (36.0, 96.3)
**Alanine aminotransferase (IU/L) [median (IQR)]**	21.0 (17.0, 28.0)	28.0 (22.0, 39.0)	17.0 (14.0, 22.0)	22.5 (18.0, 30.2)	19.0 (15.0, 24.0)	25.0 (20.0, 35.5)	21.0 (16.0, 28.0)
**Aspartate aminotransferase (IU/L) [median (IQR)]**	23.0 (19.0, 26.8)	25.0 (21.0, 32.0)	21.0 (18.0, 24.0)	23.0 (19.0, 30.0)	22.0 (18.0, 25.0)	25.0 (20.0, 31.0)	22.0 (19.0, 27.0)
**Gamma glutamyltransferase (IU/L) [median (IQR)]**	19.0 (14.0, 25.0)	28.0 (20.5, 40.5)	15.0 (11.0, 20.0)	26.0 (17.0, 43.0)	16.0 (12.0, 22.0)	28.0 (19.0, 41.0)	19.0 (14.0, 29.0)
**Platelet (10** ^**9**^ **/L) [median (IQR)]**	216.0 (185.0, 248.0)	219.0 (175.0, 256.0)	238.0 (203.0, 278.0)	263.0 (208.0, 304.0)	225.0 (193.0, 265.0)	237.0 (191.0, 282.0)	228.0 (192.0, 271.0)
**HOMA-IR [median (IQR)]**	1.59 (1.10, 2.40)	5.10 (4.08, 7.87)	1.75 (1.23, 2.49)	5.84 (4.47, 9.04)	1.67 (1.16, 2.45)	5.47 (4.20, 8.30)	2.38 (1.41, 4.36)

The detection rates of urine OPE metabolites are shown in Table 2. More than half of the subjects tested positive for five metabolites [bis (1,3-dichloro-2-propyl) phosphate (BDCPP), DBUP, bis (2-chloroethyl) phosphate (BCEP), diphenyl phosphate (DPHP), and bis (1-chloro-2-propyl) phosphate (BCPP)]. The majority of OPE metabolites were moderately linked (correlation coefficients ranged from 0.27 to 0.56) (Supplementary Table S1). In general, the individuals with NAFLD had greater amounts of urine OPE metabolites than those who without NAFLD.

**Table 2:** Detection rates (%), concentration (ng/ml) of urinary organophosphate esters in all men and women.

**OPEs**	**All**		**Male**		**Female**	
	**DR^**a**^**	**Median (25th, 75th)**	**DR**	**Median (25th, 75th)**	**DR**	**Median (25th, 75th)**
DPHP	88.8	0.69 (0.29, 1.48)	89.0	0.63 (0.29, 1.28)	88.7	0.75 (0.29, 1.64)
BDCPP	92.4	0.74 (0.30, 1.71)	94.5	0.82 (0.33, 1.75)	90.4	0.69 (0.27, 1.69)
BCPP	57.3	0.13 (0.07, 0.30)	56.9	0.12 (0.07, 0.31)	57.6	0.13 (0.07, 0.29)
BCEP	85.6	0.40 (0.16, 0.96)	87.3	0.41 (0.18, 1.04)	84.1	0.38 (0.14, 0.86)
DBUP	56.6	0.11 (0.07, 0.29)	57.8	0.12 (0.07, 0.31)	55.5	0.10 (0.07, 0.28)

^a^Detection rate, %.

### Associations of individual OPE metabolites with NAFLD

After controlling for covariates, the results showed that BCEP and BDCPP were dose-dependently positively associated with NAFLD in all males (*P*_trend_ = 0.01 for BCEP and BDCPP). By contrast, all females had only occasional meaningful relationships, except a substantial link between high DPHP concentrations and NAFLD (Table 3, Supplementary Table S2).

**Table 3:** Associations of OPE metabolites with NAFLD in all men and women.

**OPEs**	**Level**	**Male**	**Female**
		**OR (95%CI)**	**OR (95%CI)**
BCEP	C	**1.18 (1.02, 1.45)**	1.16 (0.90, 1.49)
	Q1	Ref	Ref
	Q2	0.99 (0.68, 1.65)	1.16 (0.76, 1.81)
	Q3	**1.27 (1.01, 2.35)**	1.41 (0.70, 2.83)
	Q4	**2.13 (1.15, 3.68)**	2.20 (0.77, 4.25)
	*P-*trend	< 0.01	0.26
BDCPP	C	**1.31 (1.06, 1.62)**	1.18 (0.91, 1.53)
	Q1	Ref	Ref
	Q2	1.09 (0.80, 1.68)	1.13 (0.60, 1.81)
	Q3	1.50 (0.95, 2.81)	1.37 (0.67, 2.63)
	Q4	**2.45 (1.13, 3.91)**	1.86 (0.82, 3.22)
	*P-*trend	0.02	0.45
DPHP	C	1.18 (0.91, 1.53)	1.07 (0.87, 1.31)
	Q1	Ref	Ref
	Q2	0.95 (0.68, 1.52)	0.97 (0.49, 1.62)
	Q3	1.34 (0.67, 2.68)	1.49 (0.81, 2.37)
	Q4	2.01 (0.91, 3.34)	**1.96 (1.02, 3.02)**
	*P-*trend	0.52	0.62
BCPP	C	0.96 (0.75, 1.24)	0.97 (0.75, 1.26)
	Q1	Ref	Ref
	Q2	1.05 (0.57, 1.91)	0.97 (0.65, 1.26)
	Q3	**1.89 (1.00, 3.02)**	1.22 (0.76, 2.25)
	*P-*trend	0.07	0.95
DBUP	C	1.17 (0.84, 1.64)	1.17 (0.91, 1.51)
	Q1	Ref	Ref
	Q2	1.20 (0.82, 2.15)	1.07 (0.53, 2.15)
	Q3	2.03 (0.95, 3.72)	1.25 (0.58, 2.70)
	*P-*trend	0.12	0.82

Estimates were adjusted for urinary creatinine (continuous), age (continuous), BMI (categorical), marital status (categorical), race/ethnicity (categorical), poverty income ratio (categorical), education (categorical), smoking status (binary), alcohol (binary), physical activity (binary), hypertension (binary), and diabetes (binary). Bold text indicates that the estimates are statistically significant (*P* < 0.05). C refers to “Continuous” OPE metabolites, Q1–Q4 refer to the 1st−4th quartiles of exposure. For DBUP and BCPP, Q1–Q3 indicate the 1st−3rd tertiles since their detection date < 70%.

As shown in Supplementary Table S3, the OPE metabolites and preset effect modifiers had no substantial interactions. However, the stratified analysis produced several inconsistencies. For example, BCEP, BDCPP, and NAFLD exhibited remarkable dose–response correlations in males under 60 years (*P*_trend_ = 0.02, 0.04, respectively), and high concentrations of BCEP, BDCPP, and DPHP were positively associated with NAFLD risk in men with TT levels of 426 ng/dl (*P* < 0.01). Moreover, in post-menopausal women, the highest quartile of BDCPP was significantly increased the risk of NAFLD. The connections in the men's other class and women's stratified analyzes were occasional and inconsistent (Supplementary Tables S3–S5).

### Associations of multiple OPE metabolites with NAFLD in WQS analyzes

**Figure 1:**
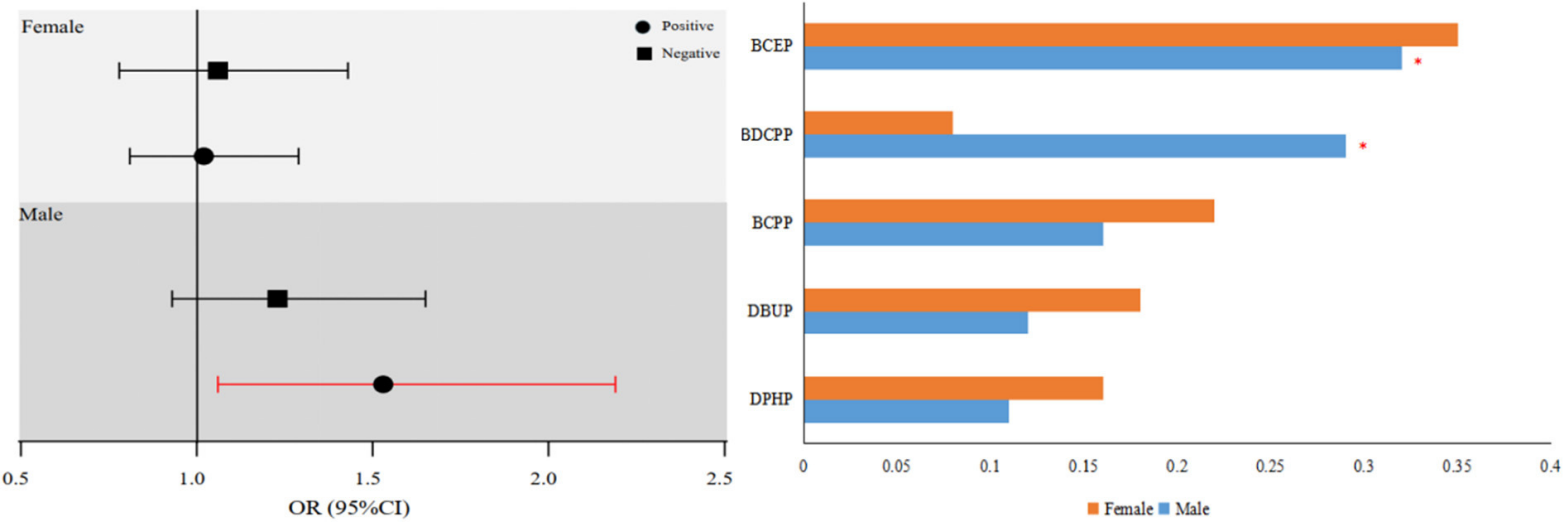
Associations of OPEs index with NAFLD in both sexes.

The OPE index was significantly increased the risk of NAFLD in all men (OR for OPE index: 1.52; 95% CI: 1.06, 2.19). In this finding, the OPE metabolites with the greatest estimated weights were BCEP (WQS weight = 0.32) and BDCPP (WQS weight = 0.29) (Figure 1).

Weighted quantile sum studies in males stratified by age and TT level revealed that the strongest correlations with the OPE combination were restricted to males < 60 years of age or TT < 432 ng/dl. In particular, men with TT < 432 ng/dl had a stable and somewhat greater favorable correlation with NAFLD than men with TT ≥432 ng/dl. A persistent favorable correlation was found for men aged 60 years. WQS analyzes in females stratified by age, TT level, and menopausal status revealed a substantial positive link between mixed OPE exposure and NAFLD in post-menopausal women, which was consistent with the results for individual OPE metabolites. However, other subgroups did not produce consistent results regarding the OPE combination. The associated weights for these connected OPE metabolites are shown in Supplementary Tables S6, S7.

### Results of sensitivity analyzes

When we employed the BKMR approach to confirm the link between mixed OPE exposure and NAFLD risk, we discovered that mixed OPE exposure was considerably positively linked with NAFLD risk, and BCEP and BDCPP were the key contributors (Supplementary Figure S2). These results were consistent with the main findings. Our sensitivity analysis also revealed that the results remained unaltered when sophisticated survey designs were not taken into account (Supplementary Table S8).

## Discussion

This study was the first to attempt to explore the relationship between urine OPE metabolites and NAFLD in people over 20 years. We discovered that these correlations differed by gender. Urinary BCEP and BDCPP levels in males were linked to increased NAFLD risk. In males, the OPE metabolite combination was likewise substantially related to NAFLD. This relationship was notably evident in males aged 60 years and those with low testosterone. By contrast, we found that mixed OPE exposure was only related to elevated NAFLD risk in post-menopausal women, and the relationships of OPE metabolites with NAFLD in women were sporadic and inconsistent.

Notably, the majority of the remarkable relationships of OPEs with NAFLD in our study were observed in men. This phenomenon might be linked to several underlying biological systems. Frog research discovered that the tissue buildup of organophosphorus flame retardants (PFRs) is very selective. Overall, the pollutant amounts in liver tissue were much greater than in other tissues. Furthermore, the pollutant concentration ratio in male frog liver tissue and other tissues was much greater than those in female frogs, indicating that male frogs have a larger metabolic capability for PFRs (21). Furthermore, the sex-dependent effects of OPEs on the hypothalamic–pituitary–thyroid axis may be important (22). Low-dose triphenyl phosphate (TPhP) exposure increased lipid-related metabolites in male mouse serum but showed no impact on female mice. In male mice, high TPhP dosages inhibit the pyruvate metabolism and citric acid cycle pathways, indicating the occurrence of aberrant lipid metabolism following TPhP exposure (23). These findings in animal tests may help explain why most NAFLD correlations are limited to males. OPEs may also have an impact on children's health. Neurodevelopment and asthma are two of the most visible negative health consequences of OPEs, with impacts on endocrine function in experimental and epidemiological investigations in adults (10). However, available research is sparse and has substantial limitations to the best of our knowledge. Few research has been conducted on the impact of OPE exposure on liver function. The effects of endocrine disruptors may occur at very low levels and early exposure during development is associated with an increased incidence of metabolism-related adverse effects such as obesity, diabetes and NAFLD (13). Several studies have linked flame retardant exposure to thyroid cancer and dose-dependent histopathological alterations in the thyroid gland, with some flame retardants competitively binding to transthyretin, a thyroxine (T4) transporter (11, 12). This results in some damage to the endocrine system. Whereas THs are essential for development, growth and metabolic activities, their importance in hepatic fatty acid and cholesterol synthesis, including metabolism, has been well-documented by studies (14). NAFLD is a complex disease and there is evidence that inflammation may precede steatosis and contribute to disease progression and may even further lead to lipid accumulation (steatosis) in hepatocytes (15). It has recently been shown that a variety of common OPFRs are able to affect inflammation-related pathways, including the JAK-STAT, TNF signaling, and PI3K-Akt pathways, and all to varying degrees in human macrophages *in vitro* (10). According to one study, males have a greater frequency and severity of NAFLD than women during their reproductive years. Moreover, post-menopausal women have greater incidences of NAFLD, indicating that estrogen has a protective effect (24). One research, however, found no statistically remarkable variation in the distribution of plasma OPEs between men and women with hypertension. This data revealed that age and gender were not factors of OPE bioaccumulation in the plasma of patients with hypertension (25).

Differences in age and hormonal status theoretically affect the risk of NAFLD, as they play a crucial role in determining the risk of NAFLD in men and women (25). It is becoming increasingly evident that once women reach menopause, they are at increasing risk of complications due to the reduced protective effects associated with estrogen. Among the protective effects of estrogen, liver fat accumulation appears to be the most important, as it plays an important role in the development of insulin resistance, atherosclerosis and cardiovascular disease (26). In recent years, there is a large body of evidence, mainly from the use of animal models, that estrogen withdrawal is indeed associated with alterations in molecular markers that favor the activity of metabolic pathways that ultimately lead to hepatic fat accumulation (27). Both basic and clinical studies support the hypothesis that estrogen has a protective effect on the development of NAFLD (28, 29) and the prevalence of NAFLD has been reported to be twice as high in post-menopausal women compared to premenopausal women, and the prevalence of NAFLD is lower in post-menopausal women receiving hormone replacement therapy (HRT) than in post-menopausal women not receiving hormone replacement therapy (30, 31). Overall, recent studies suggest that estrogen may prevent NAFLD. this hypothesis may explain our previous findings of a significant and positive association between mixed exposure to OPE and the risk of NAFLD in post-menopausal women compared to premenopausal women. At the same time, considerable differences were found across age categories, especially in men, with most of the associations with NAFLD being limited to those under 60 years of age. Furthermore, the lack of substantial association between men aged 60 years may be due to the small sample size and low statistical power of the study (*n* = 325). However, considering the arbitrary nature of age group boundaries, it is still necessary in interpreting these findings (22).

A pathogenic route may explain the link between high OPE levels and NAFLD. OPEs promote lipid accumulation in adipocytes and human hepatocytes. Transcriptomic studies in mice demonstrated that modest dosages of OPEs alter de novo fatty acid production by boosting the expression of lipogenic genes, resulting in hepatic steatosis (32–34). OPEs impair pancreatic beta-cell activity, resulting in insulin resistance in mice (35). Several investigations found that OPEs cause oxidative stress in rat hepatocytes, resulting in hepatocyte injury. OPEs were also linked to lower serum adiponectin levels in previous research (36, 37). As a result, the probable relationship between high OPE levels and NAFLD may be insulin resistance and the inflammatory response generated by the cytokines released (38). OPEs are linked to an increased risk of insulin resistance, which is linked to the pathophysiology of NAFLD (39, 40). Furthermore, OPEs can cause an inflammatory environment and oxidative stress. Higher quantities of proinflammatory cytokines have been linked to higher OPE levels (41, 42).

Previous research found that older persons are more vulnerable to NAFLD because of the impact of aging on hepatic macrophage population dynamics and polarization (43, 44). In fact, in our study, the participants over 60 years had a greater prevalence of NAFLD than those under 60 years (42.17 vs 29.14%). The elderly are a high-risk population for NALFD (45). This knowledge may help mitigate the effects of OPEs on NAFLD. Furthermore, the proportion of respondents who were 60 years old in this study was low, which may result in poor statistical power for the analysis of older people with a small sample size. Furthermore, OPEs are non-persistent pollutants; therefore, they have shorter retention durations and accumulation rates than persistent organic pollutants, such as dioxins. Older adults are likely to have different lives and use less plastic (46, 47).

Mixed OPE exposure was substantially and positively linked with NAFLD risk in post-menopausal women in our research. Although premenopausal women had more severe hepatocyte injury and inflammation than males and post-menopausal women, they had less liver fibrosis. This finding indicates the multistage influence of sex hormones on the etiology of NAFLD (48). The precise process underlying the inconsistent data is still unknown. Characterization of immune infiltrates in the livers of patients with NAFLD is needed in the future to investigate sex variations in inflammatory and cytokine profiles that may lead to fibrosis (49). Premature menopause and chronic estrogen deprivation are related to more severe liver fibrosis in post-menopausal women with NAFLD. The data suggest that estrogen protects the liver against fibrosis in patients with NAFLD. The characteristics of liver damage and inflammation in response to metabolic stress appear to be dependent on gender, puberty, and sex hormone levels (50, 51). However, more research is required in this area.

Our research offers several advantages. First, we had a reasonably high sample size, which allowed us to investigate sex differences in the connection between OPE exposure and NAFLD, as well as the possible influence of numerous major NAFLD-related characteristics when multiple covariates were controlled. Second, as a multi-pollutant model, we employed WQS regression to investigate the possible combined impacts of various OPE metabolites. This analysis provided us with more information on the OPE mixture's metabolic-disrupting effects.

Our findings, however, should be regarded with care because of the following limitations. First, our findings cannot be used to draw causal conclusions regarding the connection of OPE exposure with NAFLD because of study design constraints. Second, OPE has a very short half-life (varying from hours to days), and the exposure evaluation in the current investigation was based on a single spot of urine. Individual variability in OPE metabolite concentrations will undoubtedly induce measurement mistakes. However, such measurement mistakes might result in non-differential misclassification, skewing the genuine connection toward zero. Third, although WQS was applied to examine the mixed effects of OPE, its limitations should be recognized. WQS analysis incorporates assumptions about directional homogeneity, linearity, and the cumulative effects of different exposures, which all add uncertainty to the overall consequences of OPE. As a result, we included the BKMR approach to the sensitivity analysis to minimize this bias. Finally, although analyzes stratified by age, TT level, and menopausal status can offer useful information about the features of vulnerable populations, multiplexing may increase the likelihood of false-positive results. As a result, the current stratified analysis results are experimental. Furthermore, even after the data were corrected for various confounders, the observed relationships might be attributable to residual confounders or unmeasured variables, particularly given the presence of co-exposure pollutants, such as phthalates, which were associated with NAFLD and OPE but were measured in different environmental subsamples.

## Conclusion

In conclusion, we discovered that specific urine OPE metabolites and OPE combinations were related with an increased frequency of NAFLD in men. This connection appears to be stronger in males aged 60 years or with TT 426 ng/dl. In women, however, these correlations were sporadic and inconsistent, with the exception of an elevated risk of NAFLD linked with the OPE combination in post-menopausal women. Given the study's limitations, these findings should be evaluated with care and validated in future investigations.

## Data availability statement

The original contributions presented in the study are included in the article/Supplementary material, further inquiries can be directed to the corresponding author/s.

## Ethics statement

The studies involving human participants were reviewed and approved by the NCHS Ethic Review Board. Written informed consent to participate in this study was provided by the participants' legal guardian/next of kin.

## Author contributions

HC and WH: conceptualization, methodology, and writing—original draft. YD: software, validation, and data curation. XZ: methodology and resources. PQ: conceptualization, methodology, and supervision. JZ: conceptualization and supervision. All authors contributed to the article and approved the submitted version.

## Funding

This work was supported by the National Natural Science Foundation of China (82074386), Clinical Research Plan of SHDC (No. SHDC2020CR3095B) and Shanghai Leading Talents Program of traditional Chinese Medicine [ZY (2018-2020-RCPY-1023)]. Shanghai Three-Year Action Plan to Further Accelerate the Inheritance and Innovative Development of Traditional Chinese Medicine - (2021–2023) Cirrhosis of Liver and Ascites (Dropsy) Shanghai TCM Specialized Disease Alliance.

## Conflict of interest

The authors declare that the research was conducted in the absence of any commercial or financial relationships that could be construed as a potential conflict of interest.

## Publisher's note

All claims expressed in this article are solely those of the authors and do not necessarily represent those of their affiliated organizations, or those of the publisher, the editors and the reviewers. Any product that may be evaluated in this article, or claim that may be made by its manufacturer, is not guaranteed or endorsed by the publisher.
