# Supplementary material for: Environmental exposure to organophosphate esters and suspected non-alcoholic fatty liver disease among US adults: A mixture analysis
Source: Front Public Health. 2022 Oct 20;10:995649. doi: 10.3389/fpubh.2022.995649 (PMC9631026; doi:10.3389/fpubh.2022.995649)
Supplement: Supplementary file 1 [file Data_Sheet_1.DOCX]

**Supplementary materials**

**Environmental exposure to organophosphate esters and non-alcoholic fatty liver disease**

**among US adults: A mixture analysis**

Haisheng Chai ^a,1^, Weiye Hu ^a,1^, Yaoyao Dai ^b,1^, Xiaohan Zhu ^a^, Ping’an Qian ^b,*^, Junfeng Zhu ^c,*^

^a^ Shanghai Municipal Hospital of Traditional Chinese Medicine, Shanghai University of Traditional Chinese Medicine, Shanghai, China,

^b^ Department of Hepatology, Shanghai municipal Hospital of Traditional Chinese Medicine, Shanghai University of Traditional Chinese Medicine, Shanghai, China

^c^ Department of Hepatology, Yueyang Integrated Chinese and Western Medicine Hospital, Shanghai University of Traditional Chinese Medicine, Shanghai, China

Address corresponding to Junfeng Zhu, Department of Hepatology, Yueyang Integrated Chinese and Western Medicine Hospital, Shanghai University of Traditional Chinese Medicine, Shanghai, China. E-mail: [zhujunfeng@shutcm.edu.cn,](mailto:junjimzhang@sina.com,) or Ping’an Qian, Department of Hepatology, Shanghai municipal Hospital of Traditional Chinese Medicine, Shanghai University of Traditional Chinese Medicine, Shanghai, China. E-mail: 44129588@qq.com.

^1^ These authors contributed equally to this work.

Table S1. Pairwise Pearson correlation matrix among the selected five OPE metabolites (coefficients).

|  | BDCPP | BCEP | DPHP | BCPP | DBUP |
| --- | --- | --- | --- | --- | --- |
| BDCPP | 1.00 |  |  |  |  |
| BCEP | 0.48 | 1.00 |  |  |  |
| DPHP | 0.56 | 0.46 | 1.00 |  |  |
| BCPP | 0.37 | 0.35 | 0.32 | 1.00 |  |
| DBUP | 0.32 | 0.27 | 0.29 | 0.36 | 1.00 |

Notes: Pairwise Pearson correlation analysis was conducted based on ln-transformed OPE metabolites. All correlation coefficients were significant at 0.05.

Table S2. Regression analyses P-values after controlling for false discovery rate.

| OPEs | P FDR | |
| --- | --- | --- |
|  | Male | Female |
| BCEP | **0.002** | 0.554 |
| BDCPP | **0.026** | 0.375 |
| DPHP | 0.572 | 0.095 |
| BCPP | 0.319 | 0.717 |
| DBUP | 0.656 | 0.475 |

Table S3. P values for the interaction terms between OPE metabolites and strata of age, total testosterone and menopausal status (only for women) in both sexes.

| Sex | Group | OPEs | MetS |
| --- | --- | --- | --- |
| Men | Age  group | BCEP | **0.01*** |
|  |  | BCPP | 0.16 |
|  |  | BDCPP | 0.56 |
|  |  | DBUP | 0.16 |
|  |  | DPHP | 0.24 |
|  | TT  level | BCEP | 0.60 |
|  |  | BCPP | 0.10 |
|  |  | BDCPP | 0.49 |
|  |  | DBUP | 0.61 |
|  |  | DPHP | 0.95 |
| Women | Age  group | BCEP | 0.19 |
|  |  | BCPP | 0.93 |
|  |  | BDCPP | 0.90 |
|  |  | DBUP | 0.49 |
|  |  | DPHP | 0.35 |
|  | Menopausal status | BCEP | 0.56 |
|  |  | BCPP | 0.47 |
|  |  | BDCPP | **0.02*** |
|  |  | DBUP | 0.46 |
|  |  | DPHP | 0.22 |
|  | TT  group | BCEP | 0.81 |
|  |  | BCPP | 0.26 |
|  |  | BDCPP | 0.66 |
|  |  | DBUP | 0.42 |
|  |  | DPHP | **0.04*** |

Table S4. Associations of OPE metabolites with NAFLD by age group stratified by gender.

| Age group | OPEs | Level | Male | Female |
| --- | --- | --- | --- | --- |
|  |  |  | OR (95%CI) | OR (95%CI) |
| <60 yrs | BCEP | C | **1.25 (1.02,1.53)** | 1.12 (0.75,1.69) |
|  |  | Q1 |  |  |
|  |  | Q2 | 1.16 (0.56,2.41) | 0.99 (0.43,2.27) |
|  |  | Q3 | 1.26 (0.84,2.59) | 1.10 (0.45,2.70) |
|  |  | Q4 | **2.13 (1.15,3.76)** | 1.37 (0.71,2.63) |
|  |  | *P*-trend | **0.02** | 0.27 |
|  | BDCPP | C | 1.18 (0.91,1.53) | 1.33 (0.94,1.90) |
|  |  | Q1 |  |  |
|  |  | Q2 | 1.34 (0.87,2.68) | 1.13 (0.62,2.07) |
|  |  | Q3 | **1.71 (1.00,3.34)** | 1.20 (0.54,2.70) |
|  |  | Q4 | **1.86 (1.09,4.22)** | **1.30 (1.00,1.70)** |
|  |  | *P-*trend | **＜0.01** | 0.07 |
|  | DPHP | C | 1.30 (0.98,1.72) | 1.20 (0.89,1.63) |
|  |  | Q1 |  |  |
|  |  | Q2 | 1.13 (0.60,2.13) | 1.01 (0.46,2.22) |
|  |  | Q3 | 1.19 (0.56,2.56) | 1.09 (0.54,2.21) |
|  |  | Q4 | 1.61 (0.81,3.18) | 1.40 (0.59,3.94) |
|  |  | *P*-trend | 0.57 | 0.65 |
|  | BCPP | C | 1.09 (0.83,1.45) | 1.13 (0.90,1.43) |
|  |  | Q1 |  |  |
|  |  | Q2 | 0.97 (0.49,1.62) | 1.13 (0.55,2.32) |
|  |  | Q3 | 1.18 (0.64,2.15) | 1.65 (0.80,3.40) |
|  |  | *P*-trend | 0.16 | 0.19 |
|  | DBUP | C | 1.17 (0.91,1.51) | 1.09 (0.86,1.39) |
|  |  | Q1 |  |  |
|  |  | Q2 | 1.02 (0.55,1.68) | 1.04 (0.56,1.91) |
|  |  | Q3 | 1.24 (0.69,2.22) | 1.35 (0.73,2.48) |
|  |  | *P*-trend | 0.72 | 0.36 |
| ≥60 yrs | BCEP | C | 1.17 (0.84,1.64) | 0.95 (0.66,1.37) |
|  |  | Q1 |  |  |
|  |  | Q2 | 1.13 (0.59,3.24) | 0.93 (0.32,2.67) |
|  |  | Q3 | 1.20 (0.65,3.67) | 1.53 (0.48,4.85) |
|  |  | Q4 | 1.32 (0.71,4.69) | 1.03 (0.26,4.13) |
|  |  | *P*-trend | 0.42 | 0.51 |
|  | BDCPP | C | 1.33 (0.94,1.87) | 1.05 (0.70,1.57) |
|  |  | Q1 |  |  |
|  |  | Q2 | 0.95 (0.36,2.49) | 1.04 (0.34,3.13) |
|  |  | Q3 | 1.13 (0.59,3.26) | 0.87 (0.31,2.43) |
|  |  | Q4 | 2.08 (0.75,6.71) | 2.29 (0.65,8.15) |
|  |  | *P*-trend | 0.15 | 0.87 |
|  | DPHP | C | 1.15 (0.72,1.83) | 1.24 (0.83,1.84) |
|  |  | Q1 |  |  |
|  |  | Q2 | 1.70 (0.43,6.78) | 0.84 (0.27,2.54) |
|  |  | Q3 | **2.44 (1.05,8.21)** | 1.23 (0.38,3.95) |
|  |  | Q4 | 2.79 (0.80,8.33) | 1.01 (0.33,3.11) |
|  |  | *P*-trend | **0.05** | 0.59 |
|  | BCPP | C | 0.98 (0.62,1.56) | 0.94 (0.59,1.50) |
|  |  | Q1 |  |  |
|  |  | Q2 | 1.24 (0.44,3.54) | 1.58 (0.65,3.80) |
|  |  | Q3 | 0.85 (0.33,2.17) | 2.46 (0.82,7.43) |
|  |  | *P*-trend | 0.76 | 0.61 |
|  | DBUP | C | 1.03 (0.62,1.72) | 1.11 (0.67,1.84) |
|  |  | Q1 |  |  |
|  |  | Q2 | **2.62 (1.01,4.98)** | 1.32 (0.44,3.97) |
|  |  | Q3 | 1.57 (0.39,6.26) | 1.07 (0.41,2.80) |
|  |  | *P*-trend | 0.11 |  |

Table S5. Associations of OPE metabolites with NAFLD by total testosterone level stratified by gender.

| Subgroup | OPEs | Level | Male | Female |
| --- | --- | --- | --- | --- |
|  |  |  | OR (95%CI) | OR (95%CI) |
| HTT | BCEP | C | 1.28 (0.87,1.89) | 1.04 (0.79,1.37) |
|  |  | Q1 |  |  |
|  |  | Q2 | 1.05 (0.43,2.52) | 0.95 (0.40,2.27) |
|  |  | Q3 | 1.35 (0.44,4.21) | 1.01 (0.31,3.29) |
|  |  | Q4 | 1.77 (0.49,6.47) | 1.18 (0.40,3.50) |
|  |  | *P*-trend | 0.27 | 0.86 |
|  | BDCPP | C | 1.13 (0.85,1.50) | 1.18 (0.85,1.65) |
|  |  | Q1 |  |  |
|  |  | Q2 | 0.79 (0.27,2.30) | 1.26 (0.43,376) |
|  |  | Q3 | 1.11 (0.48,2.57) | 0.90 (0.33,2.47) |
|  |  | Q4 | 2.09 (0.83,5.28) | 1.39 (0.45,4.26) |
|  |  | *P-*trend | 0.41 | 0.18 |
|  | DPHP | C | 1.19 (0.87,1.63) | 1.08 (0.79,1.48) |
|  |  | Q1 |  |  |
|  |  | Q2 | 1.56 (0.69,3.52) | 1.28 (0.39,4.25) |
|  |  | Q3 | **2.73 (1.08,6.21)** | 1.17 (0.50,2.78) |
|  |  | Q4 | 3.05 (0.88,10.72) | 1.36 (0.62,3.30) |
|  |  | *P*-trend | 0.17 | 0.41 |
|  | BCPP | C | 1.07 (0.81,1.42) | 0.97 (0.72,1.30) |
|  |  | Q1 |  |  |
|  |  | Q2 | 0.90 (0.32,2.56) | 1.24 (0.61,2.53) |
|  |  | Q3 | 1.14 (0.42,3.09) | 1.14 (0.58,2.23) |
|  |  | *P*-trend | 0.59 | 0.69 |
|  | DBUP | C | 0.96 (0.63,1.48) | 0.99 (0.72,1.36) |
|  |  | Q1 |  |  |
|  |  | Q2 | 0.71 (0.29,1.75) | 0.95 (0.43,2.06) |
|  |  | Q3 | 1.22 (0.44,3.35) | 1.51 (0.71,3.25) |
|  |  | *P*-trend | 0.31 | 0.26 |
| LTT | BCEP | C | **1.32 (1.05,1.70)** | 1.26 (0.87,1.82) |
|  |  | Q1 |  |  |
|  |  | Q2 | 1.16 (0.51,2.63) | 1.18 (0.56,2.52) |
|  |  | Q3 | 1.58 (0.70,3.59) | 0.97 (0.42,2.27) |
|  |  | Q4 | **2.65 (1.12,7.59)** | 2.17 (0.79,5.97) |
|  |  | *P*-trend | **0.02** | 0.75 |
|  | BDCPP | C | **1.54 (1.10,2.16)** | 1.13 (0.86,1.50) |
|  |  | Q1 |  |  |
|  |  | Q2 | 1.23 (0.51,2.96) | 1.17 (0.47,2.89) |
|  |  | Q3 | **1.92 (1.06,4.30)** | 1.71 (0.91,3.75) |
|  |  | Q4 | **2.81 (1.27,5.59)** | **2.57 (1.00,6.91)** |
|  |  | *P*-trend | **<0.01** | 0.07 |
|  | DPHP | C | 1.14 (0.86,1.51) | 0.99 (0.75,1.31) |
|  |  | Q1 |  |  |
|  |  | Q2 | **1.67 (1.01,2.38)** | 0.87 (0.39,1.95) |
|  |  | Q3 | 1.24 (0.62,2.97) | 1.06 (0.31,2.25) |
|  |  | Q4 | **1.82 (1.09,4.79)** | 1.51 (0.56,4.11) |
|  |  | *P*-trend | **0.04** | 0.47 |
|  | BCPP | C | 1.13 (0.82,1.56) | 1.06 (0.76,1.49) |
|  |  | Q1 |  |  |
|  |  | Q2 | 1.08 (0.45,2.61) | 1.22 (0.54,2.75) |
|  |  | Q3 | 1.77 (0.92,3.63) | 1.56 (0.74,3.29) |
|  |  | *P*-trend | 0.65 | 0.27 |
|  | DBUP | C | 0.96 (0.68,1.34) | 1.02 (0.69,1.56) |
|  |  | Q1 |  |  |
|  |  | Q2 | 0.93 (0.43,1.99) | 1.18 (0.56,2.50) |
|  |  | Q3 | 1.22 (0.57,2.62) | 0.89 (0.45,1.77) |
|  |  | *P*-trend | 0.22 | 0.61 |

Table S6. Associations of OPE metabolites with NAFLD by menopausal status in women.

| OPEs | Level | Premenopausal | Postmenopausal |
| --- | --- | --- | --- |
|  |  | OR (95%CI) | OR (95%CI) |
| BCEP | C | 1.15 (0.93,1.41) | 1.37 (0.96,1.96) |
|  | Q1 | Ref | Ref |
|  | Q2 | 0.90 (0.44,1.83) | 1.07 (0.51,2.22) |
|  | Q3 | 1.16 (0.55,2.46) | 1.26 (0.67,3.51) |
|  | Q4 | 1.60 (0.75,3.62) | 2.04 (0.89,4.67) |
|  | *P-*trend | 0.25 | 0.07 |
| BDCPP | C | 1.20 (0.94,1.52) | **1.09 (1.01,1.36)** |
|  | Q1 | Ref | Ref |
|  | Q2 | 1.15 (0.53,2.48) | 1.31 (0.65,2.62) |
|  | Q3 | 1.51 (0.71,3.21) | **1.81 (1.00,4.30)** |
|  | Q4 | 2.26 (0.95,5.35) | **2.06 (1.07,4.42)** |
|  | *P-*trend | 0.36 | **0.05** |
| DPHP | C | 1.02 (0.81,1.29) | 1.26 (0.71,2.17) |
|  | Q1 | Ref | Ref |
|  | Q2 | 0.92 (0.46,1.82) | 1.12 (0.29,4.23) |
|  | Q3 | 1.04 (0.47,2.30) | 1.57 (0.50,4.98) |
|  | Q4 | 1.55 (0.64,3.75) | 2.17 (0.72,6.05) |
|  | *P-*trend | 0.62 | 0.57 |
| BCPP | C | 0.92 (0.72,1.18) | 1.11 (0.63,1.94) |
|  | Q1 | Ref | Ref |
|  | Q2 | 0.91 (0.49,1.68) | 1.76 (0.85,5.12) |
|  | Q3 | 1.35 (0.72,2.52) | **2.16 (1.01,7.51)** |
|  | *P-*trend | 0.16 | 0.06 |
| DBUP | C | 1.06 (0.71,1.58) | 1.09 (0.61,1.95) |
|  | Q1 | Ref | Ref |
|  | Q2 | 1.17 (0.60,2.28) | 1.16 (0.35,3.62) |
|  | Q3 | 1.82 (0.99,3.37) | 1.62 (0.65,5.71) |
|  | *P-*trend | 0.27 | 0.72 |

Table S7. Associations between OPEs index and sex hormone indicators by sex-age and sex-puberty status after further adjusting for daily total intake of fat in participants of 6-19 years old in NHANES 2013-2014.

|  | Groups | WQS  direction | Male | Female |
| --- | --- | --- | --- | --- |
|  |  |  | OR (95%CI) | OR (95%CI) |
| TT level | HTT | N | 1.11 (0.64,1.92) | 1.12 (0.59,2.12) |
|  |  | P | 1.35 (0.76,2.32) | 1.39 (0.82,2.36) |
|  | LTT | N | 0.98 (0.66,1.46) | 0.72 (0.49,1.08) |
|  |  | P | **1.67 (1.06,2.55)** | 1.13 (0.76,1.68) |
| Age group | ≥60 years | N | 0.97 (0.58,1.59) | 0.87 (0.56,1.48) |
|  |  | P | 1.52 (0.76,3.01) | 1.05 (0.51,2.17) |
|  | <60 years | N | 1.15 (0.86,1.54) | 0.86 (0.57,1.28) |
|  |  | P | **1.61 (1.12,2.37)** | 1.37 (0.89,2.11) |
| Menopause | No (n=427) | N | - | 0.95 (0.65,1.39) |
|  |  | P | **-** | 1.28 (0.82,2.00) |
|  | Yes (n=156) | N | - | 0.75 (0.36,1.68) |
|  |  | P | - | **1.48 (1.06,2.06)** |

Table S8. Weights of OPE metabolites in WQS regression for the associations with NAFLD by different groups among 6-19 years old participants in NHANES 2011-2014.

| Subgroup | Direction | OPE  metabolites | Male | Female |
| --- | --- | --- | --- | --- |
| All | Negative | BCEP | 0.06 | 0.23 |
|  |  | BCPP | 0.38 | 0.50 |
|  |  | BDCPP | 0.19 | 0.13 |
|  |  | DBUP | 0.00 | 0.06 |
|  |  | DPHP | 0.37 | 0.08 |
|  | Positive | BCEP | **0.32** | 0.35 |
|  |  | BCPP | 0.16 | 0.22 |
|  |  | BDCPP | **0.29** | 0.08 |
|  |  | DBUP | 0.12 | 0.18 |
|  |  | DPHP | 0.11 | 0.16 |
| HTT | Negative | BCEP | 0.51 | 0.42 |
|  |  | BCPP | 0.01 | 0.17 |
|  |  | BDCPP | 0.17 | 0.04 |
|  |  | DBUP | 0.08 | 0.22 |
|  |  | DPHP | 0.23 | 0.14 |
|  | Positive | BCEP | 0.31 | 0.31 |
|  |  | BCPP | 0.31 | 0.31 |
|  |  | BDCPP | 0.00 | 0.00 |
|  |  | DBUP | 0.37 | 0.37 |
|  |  | DPHP | 0.01 | 0.01 |
| LTT | Negative | BCEP | 0.55 | 0.23 |
|  |  | BCPP | 0.12 | 0.26 |
|  |  | BDCPP | 0.11 | 0.22 |
|  |  | DBUP | 0.19 | 0.04 |
|  |  | DPHP | 0.04 | 0.25 |
|  | Positive | BCEP | **0.35** | 0.03 |
|  |  | BCPP | 0.06 | 0.18 |
|  |  | BDCPP | 0.27 | 0.14 |
|  |  | DBUP | 0.12 | 0.14 |
|  |  | DPHP | 0.20 | **0.51** |
| ≥ 60 years | Negative | BCEP | 0.26 | 0.07 |
|  |  | BCPP | 0.22 | 0.12 |
|  |  | BDCPP | 0.03 | 0.17 |
|  |  | DBUP | 0.46 | 0.43 |
|  |  | DPHP | 0.03 | 0.20 |
|  | Positive | BCEP | 0.78 | 0.05 |
|  |  | BCPP | 0.00 | 0.26 |
|  |  | BDCPP | 0.22 | 0.23 |
|  |  | DBUP | 0.00 | 0.37 |
|  |  | DPHP | 0.00 | 0.09 |
| < 60 years | Negative | BCEP | 0.17 | 0.22 |
|  |  | BCPP | 0.15 | 0.07 |
|  |  | BDCPP | 0.12 | 0.48 |
|  |  | DBUP | 0.15 | 0.00 |
|  |  | DPHP | 0.41 | 0.23 |
|  | Positive | BCEP | **0.57** | 0.32 |
|  |  | BCPP | 0.10 | 0.15 |
|  |  | BDCPP | 0.00 | 0.03 |
|  |  | DBUP | 0.32 | 0.20 |
|  |  | DPHP | 0.00 | 0.29 |
| No | Negative | BCEP | - | 0.39 |
|  |  | BCPP | - | 0.40 |
|  |  | BDCPP | - | 0.19 |
|  |  | DBUP | - | 0.01 |
|  |  | DPHP | - | 0.01 |
|  | Positive | BCEP | - | 0.03 |
|  |  | BCPP | - | 0.41 |
|  |  | BDCPP | - | 0.07 |
|  |  | DBUP | - | 0.18 |
|  |  | DPHP | - | 0.31 |
| Yes | Negative | BCEP | - | 0.12 |
|  |  | BCPP | - | 0.08 |
|  |  | BDCPP | - | 0.51 |
|  |  | DBUP | - | 0.17 |
|  |  | DPHP | - | 0.12 |
|  | Positive | BCEP | - | 0.01 |
|  |  | BCPP | - | **0.60** |
|  |  | BDCPP | - | 0.23 |
|  |  | DBUP | - | 0.06 |
|  |  | DPHP | - | 0.10 |

|  | NAFLD |
| --- | --- |
| Gender | -0.096** |
| Age | 0.159** |
| BMI | 0.538* |
| PIR | -0.078** |
| Marital status | -0.098** |
| Education | -0.077* |
| Race/ethnicity | -0.179** |
| Smoking status | 0.009 |
| Alcohol | 0.007 |
| Physical activity | -0.162** |
| Hypertension | 0.254** |
| Diabetes | 0.287** |
| Creatinine | 0.132*** |
| Total cholesterol | 0.012 |

Table S9. Pearson's correlation matrix (coefficients) between the selected covariates and NAFLD.


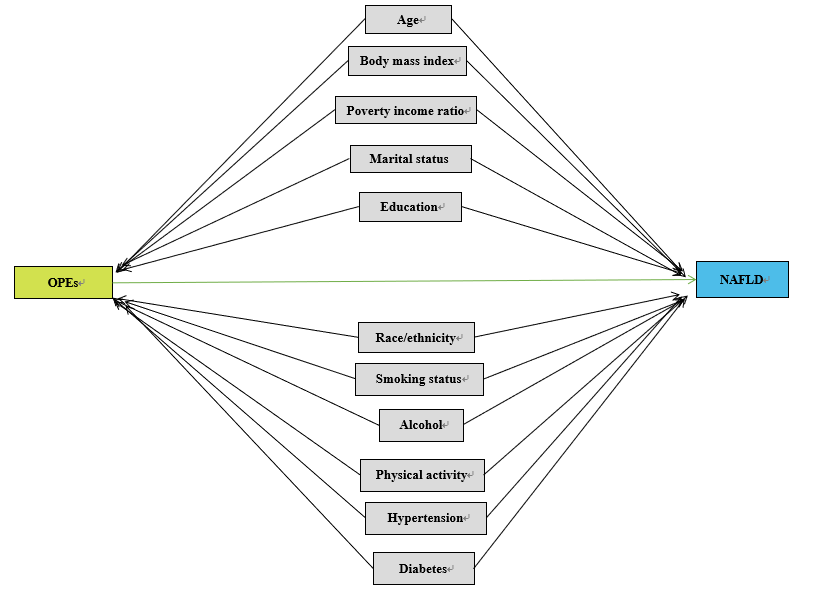


Figure S1. DAG plot.

**A B**


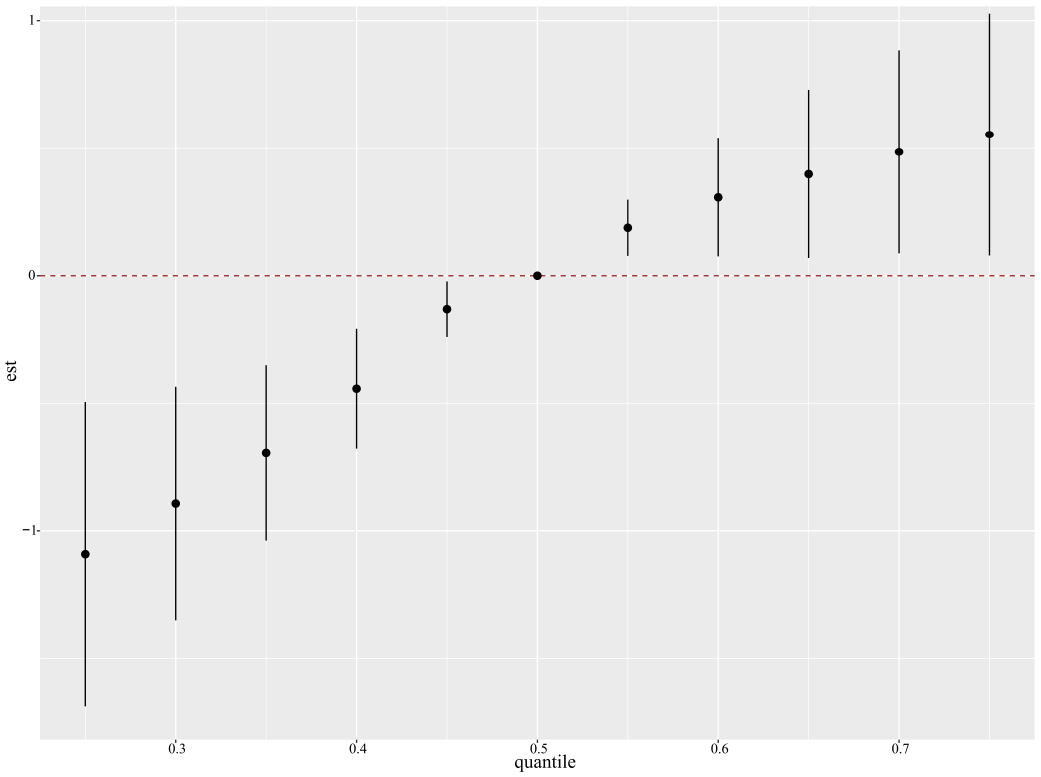

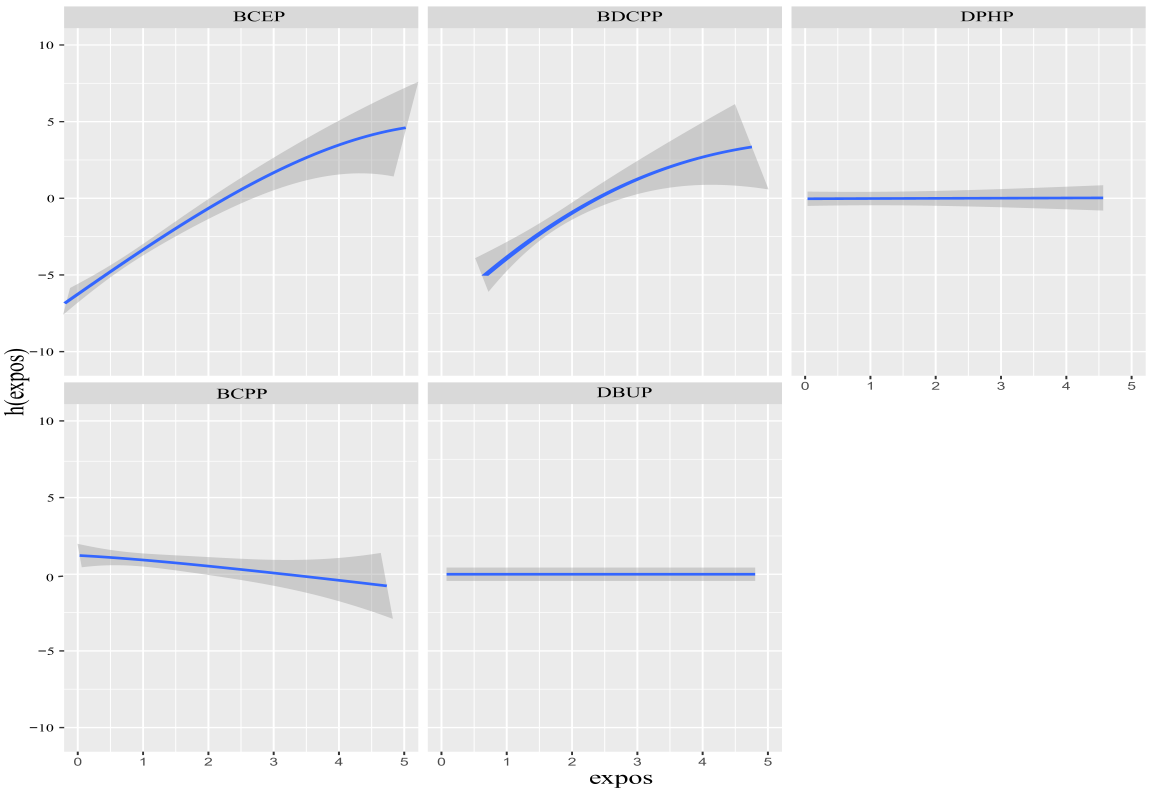


Figure S2 Overall joint associations of OPEs mixture with the NAFLD risk estimated by Bayesian kernel machine regression (BKMR) (A). Univariate exposure-response (ER) relationship of individual OPEs with the risk of NAFLD (B). All estimates were adjusted for urinary creatinine (continuous), age (continuous), BMI (categorical), marital status (categorical), race/ethnicity (categorical), poverty income ratio (categorical), education (categorical), smoking status (binary), alcohol (binary), physical activity (binary), hypertension (binary) and diabetes (binary). The boundaries of the grey areas represented the 95% CIs of ER relationsh
